# Chemolipiodolization with or without embolization in transcatheter arterial chemoembolization combined with radiofrequency ablation for hepatocellular carcinoma—propensity score matching analysis

**DOI:** 10.18632/oncotarget.8897

**Published:** 2016-04-21

**Authors:** Feng Shi, Liang Zhang, Shuai Li, Cai-Jin Lin, Lu-Jun Shen, Chao-Feng Li, Mei Jie, Zhi-Wen Li, Pei-Hong Wu

**Affiliations:** ^1^ State Key Laboratory of Oncology in South China, Collaborative Innovation Center of Cancer Medicine, Sun Yat-sen University Cancer Center, Guangzhou, China; ^2^ The First Affiliated Hospital of Zhengzhou University, Zhengzhou, China; ^3^ Zhong-shan School of Medicine, Sun Yat-Sen University, Guangzhou, China

**Keywords:** transcatheter arterial chemoembolization, radiofrequency ablation, hepatocellular carcinoma, Milan criteria, propensity score matching analysis

## Abstract

To retrospectively compare the outcome of chemolipiodolization with or without embolization in transcatheter arterial chemoembolization (TACE) combined with radiofrequency ablation (RFA) in Patients with hepatocellular carcinoma (HCC) within the Milan criteria. From August 2002 to December 2014, 112 patients (median age, 56.7 years; age range, 22–80 years; 97 men, 15 women) underwent TACE with gelatin sponge particle embolization, and 125 patients (median age, 56.6 years; age range, 23–82 years; 109 men, 16 women) underwent TACE without embolization. RFA was performed within 2 weeks after the TACE. Cumulative overall survival (OS) and disease-free survival (DFS) rates were compared before and after propensity score matching. Before matching, the 1-, 3-, and 5-year OS rate were 96%, 80%, and 62% for embolization group and 94%, 76%, and 59% for non-embolization group. The 1-, 3-, and 5-year DFS rate were 77%, 38%, and 30% for embolization group and 75%, 35%, and 26% for non-embolization group. After matching, the 1-, 3-, and 5-year OS rate were 97%, 82%, and 62% for embolization group and 92%, 74%, and 56% for non-embolization group. The 1-, 3-, and 5-year DFS rate were 79%, 36%, and 30% for embolization group and 74%, 33%, and 26% for non-embolization group. There were no significant difference in OS and DFS rates between the two groups before matching (P =0.999 and P =0.654) and after matching (P =0.951 and P =0.670). In conclusion, embolization in TACE combined with RFA could not improve the survival for patients with HCC within the Milan criteria.

## INTRODUCTION

Hepatocellular carcinoma (HCC) is the sixth most common malignant tumor worldwide and the second leading cause of cancer-related death, with China alone accounting for almost 50% of the total number of cases and deaths [1]. Patients within the Milan criteria [2] (single nodule with size ≤5 cm or up to three nodules with tumor size ≤3 cm without vascular invasion or extrahepatic metastasis) are optimal candidates for liver transplantation. Despite many studies showing excellent outcome for liver transplantation, there are several limitations such as high risks and costs, the need for organ donors, and the need for lifelong immunosuppression. Therefore, patients with preserved liver function (normal portal pressure/ bilirubin) may also qualify for other curative treatments, such as surgical resection or radiofrequency ablation (RFA). Although surgical resection is considered the main treatment in patients with early HCC, surgical resection increases the risk of postoperative liver failure compared with RFA.

Radiofrequency ablation (RFA) is generally considered one of the first-line treatments for early stage HCC, particularly for patients with impaired liver function. It is considered comparable to hepatic resection in terms of overall survival, as evidenced by numerous studies. While, tumor recurrence due to incomplete ablation is a negative prognostic factor for patient survival [3–5]. Thus, to minimize recurrence rate and prolong survival, the combined use of transcatheter arterial chemoembolization (TACE) with RFA is applicable [6]. After TACE procedure, the main artery supplying the tumor may be occluded. The subsequent RFA is more effective owing to minimized heat loss by convection [7]. Moreover, TACE combined with RFA has shown synergistic cytotoxic effects in HCC [6, 8]. Studies have shown that TACE combined with RFA had better efficacy than that of RFA alone, especially for medium-sized HCCs [9–10].

In classic TACE, embolization is performed after chemolipiodolization, which is a common practice in china and other countries for the treatment of HCC [9–13]. However, some clinicians are of the opinion that chemolipiodolization alone plays a major role and embolization does not improve the survival [14, 15], while some others have contrary opinions [16, 17]. In combined TACE and RFA procedure, the value of embolization had not been tested before. As a result, treatment protocols vary among our centers, owing to diverse clinical opinions. The purpose of our retrospective study was to compare the outcome of TACE with or without embolization combined with RFA in patients with HCC within the Milan criteria.

## RESULTS

### Baseline caracteristics of embolization and non-embolization groups

The median follow-up periods were 44.2 months (range, 7.7 – 123.7 months) in the embolization group and 40.7 months (range, 1.9 – 146.9 months) in the non-embolization group. The baseline patient characteristics for the two groups are shown in Table 1. A larger proportion of patients in the embolization group had larger tumors (P = 0.015). In contrast, patients in the non- embolization group were more frequently classified as having Child–Pugh class B disease (P = 0.016). Other independent variables mentioned above did not show significant intergroup differences.

**Table 1:** Baseline patient characteristics before propensity score matching

Variable	Embolization Group (n=112)	Non-embolization Group (n=125)	P Value
Age, years*	56.7 (22-80)	56.6 (23-82)	0.447
Sex			0.999
Male	97 (87)	109 (87)	
Female	15 (13)	16 (13)	
Diabetes mellitus			0.336
Yes	23 (21)	20 (16)	
No	89 (79)	105 (84)	
Hypertension			0.159
Yes	25 (22)	19 (15)	
No	87 (78)	106 (85)	
Anti-HCV status			0.874
Positive	108 (96)	121 (97)	
Negative	4 (4)	4 (3)	
HBsAg			0.333
Positive	11 (10)	8 (6)	
Negative	101 (90)	117 (94)	
AFP, ng/mL			0.186
>200	33 (29)	47 (38)	
≤200	79 (71)	78 (62)	
No. of nodules			0.914
Solitary	76 (68)	84 (67)	
Multiple	36 (32)	41 (33)	
Size of main tumor, cm			0.015
>2	96 (86)	91 (73)	
≤2	16 (14)	34 (27)	
Child-Pugh class			0.016
A	107 (96)	108 (86)	
B	5 (4)	17 (14)	
ALT, μ/L			0.682
>40	49 (44)	58 (46)	
≤40	63 (56)	67 (54)	
Platelet count, 10E9/L			0.508
≥100	71 (63)	74 (59)	
<100	41 (37)	51 (41)	

Note: Unless otherwise indicated, data are number of patients, with percentages in parentheses. anti-HCV = antibody to hepatitis C virus, HBsAg = hepatitis B surface antigen. AFP = a-fetoprotein, ALT = alanine aminotransferase,

* Data are medians, and data in parentheses are the range.

### Technical success of RFA

Technical success was achieved in 100 of 112 (89.2%) patients in the embolization group after the first RFA session. For the ten (9.8%) patients with residual viable tumor, technical success was achieved after an additional session of RFA. One (0.9%) patient achieve technical success after three RFA sessions. For the non-embolization group, one RFA session was performed in 112 (89.6%) patients, two RFA sessions were performed in 12 (9.6%) patients, and three RFA sessions were performed in one (0.8%) patient.

### Comparison of OS and DFS rates between the groups before propensity score matching

#### OS rate

**Figure 1:**
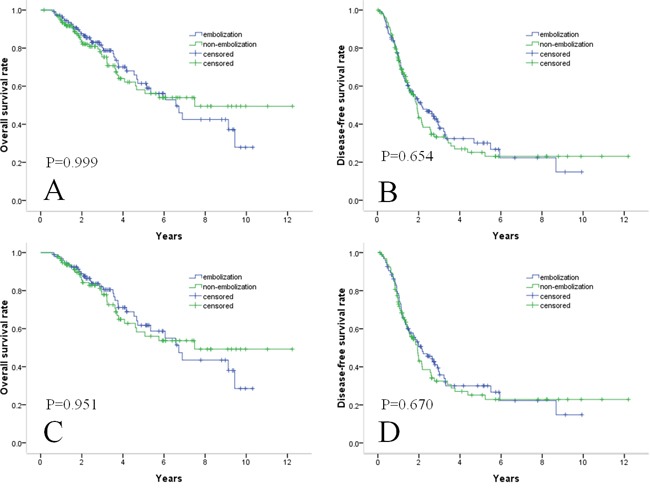
Survival curves in patients with HCC within Milan criteria who underwent TACE with or without embolization combined with RF Cumulative OS curves **A.** and cumulative DFS curves **B.** before propensity score matching. Cumulative OS curves **C.** and cumulative DFS curves **D.** after propensity score matching. There were no significant differences in survival outcomes either before or after propensity score matching.

During the follow-up period, 37 of 112 (33.0%) patients in the embolization group and 37 of 125 (29.6%) patients in the non-embolization group died. The estimated OS rates at 1, 3, and 5 years were 96%, 80%, and 62%, respectively, for the 112 patients in the embolization group and 94%, 76%, and 59%, respectively, for the 125 patients in the non-embolization group (Figure 1A). The differences between the groups were not significant (P = 0.999). In all patients, larger tumor size (>2 cm; P = 0.013), Child–Pugh class B disease (P = 0.026), and higher ALT levels (>40 IU/L; P = 0.040) were significantly associated with OS in univariate analyses (Table 2). In multivariate analyses, higher AFP levels (>200 ng/mL; P = 0.010), larger tumor size (>2 cm; P = 0.012), Child–Pugh class B disease (P = 0.031), and higher ALT levels (>40 IU/L; P = 0.022) were independent predictors of OS. However, treatment with embolization as opposed to treatment without embolization was not an independent risk factor for OS (P = 0.999) (Table 2).

**Table 2:** Univariate and multivariate analyses of prognostic factors for overall survival

Variable	Univariate Analysis			Multivariate Analysis		
	HR	95% CI	P Value	HR	95% CI	*P* Value
With embolization	1.000	0.634,1.579	0.999	0.944	0.582,1.530	0.816
Age>60	1.205	0.762,1.906	0.424	1.400	0.825,2.377	0.212
Male sex	1.188	0.514,2.746	0.687	1.251	0.523,2.996	0.615
Diabetes mellitus	1.472	0.854,2.538	0.164	1.095	0.590,2.030	0.774
Hypertension	0.927	0.508,1.689	0.804	0.744	0.388,1.427	0.373
HBsAg positive	0.743	0.347,1.621	0.455	0.670	0.256,1.753	0.414
Anti-HCV positive	1.194	0.375,3.801	0.764	1.145	0.277,4.737	0.852
AFP>200 ng/mL	1.525	0.964,2.414	0.071	1.860	1.158, 2.989	0.010
multiple nodules	1.542	0.972,2.445	0.066	1.567	0.947,2.592	0.080
Size of main tumor>2cm	2.344	1.199,4.586	0.013	2.447	1.216,4.923	0.012
Child-Pugh class B	2.337	1.108,4.930	0.026	2.407	1.083,5.352	0.031
ALT level >40 IU/L	1.618	1.023,2.558	0.040	1.751	1.084,2.829	0.022
Platelet count<10E9/L	1.545	0.968,2.468	0.068	1.523	0.917,2.528	0.104

Note: anti-HCV = antibody to hepatitis C virus, HBsAg = hepatitis B surface antigen. AFP = a-fetoprotein, ALT = alanine aminotransferase, HR = hazard ratio.

#### DFS rate

A total of 69 (61.6%) patients in the embolization group and 74 (59.2%) patients in the non-embolization group showed recurrence. The 69 recurrences in patients with embolization were intrahepatic recurrences (8 local tumor progressions, 58 distant intrahepatic tumor progressions), and three involved an extrahepatic (lymph and lung) metastases. The 74 recurrences in patients without embolization were intrahepatic recurrences (9 local tumor progressions, 63 distant intrahepatic recurrences), and two involved extrahepatic (lung) metastases. The cumulative DFS rates at 1, 3, and 5 years were 77%, 38%, and 30%, respectively, for the embolization group and 75%, 35%, and 26%, respectively, for the non-embolization group (Figure 1B). DFS rates between the two groups did not differ significantly (P = 0.654). With regard to DFS, only multiple nodules (P = 0.002) was identified as an independent factor at univariate analysis. At multivariate analysis, multiple nodules (P = 0.009), and larger tumor size (>2 cm; P = 0.028) were significant factors (Table 3).

**Table 3:** Univariate and multivariate analyses of prognostic factors for disease-free survival

Variable	Univariate Analysis			Multivariate Analysis		
	HR	95% CI	P Value	HR	95% CI	*P* Value
With embolization	1.078	0.776,1.498	0.655	1.060	0.755,1.488	0.738
Age>60	0.808	0.577,1.133	0.217	0.810	0.556,1.180	0.272
Male sex	1.209	0.683,2.139	0.515	1.077	0.590,1.967	0.808
Diabetes mellitus	1.193	0.778,1.829	0.418	1.128	0.706,1.802	0.614
Hypertension	0.801	0.508,1.262	0.339	0.897	0.552,1.458	0.662
HBsAg positive	1.234	0.627,2.428	0.544	0.932	0.443,1.963	0.854
Anti-HCV positive	0.230	0.158,1.559	0.469	0.579	0.163,2.059	0.399
AFP>200 ng/mL	1.195	0.850, 1.681	0.306	1.256	0.886,1.781	0.201
multiple nodules	1.719	1.227,2.406	0.002	1.616	1.127,2.317	0.009
Size of main tumor>2cm	1.489	0.983,2.256	0.060	1.628	1.053,2.518	0.028
Child-Pugh class B	1.411	0.811,2.454	0.223	1.491	0.835,2.663	0.177
ALT level >40 IU/L	1.198	0.862,1.665	0.281	1.168	0.833,1.638	0.367
Platelet count<10E9/L	0.918	0.650,1.295	0.625	0.833	0.575,1.206	0.334

Note: anti-HCV = antibody to hepatitis C virus, HBsAg = hepatitis B surface antigen. AFP = a-fetoprotein, ALT = alanine aminotransferase, HR = hazard ratio.

### Comparison of OS and DFS rates between the groups after propensity score matching

A total of 95 patients from each group were matched by applying 1:1 propensity score matching. All the independent variables were well matched between the two groups (Table 4). The estimated OS rates at 1, 3, and 5 years were 97%, 82%, and 62%, respectively, for patients in the embolization group and 92%, 74%, and 56%, respectively, for patients in the non-embolization group (Figure 1C). The cumulative DFS rates at 1, 3, and 5 years were 79%, 36%, and 30%, respectively, for the embolization group and 74%, 33%, and 26%, respectively, for the non-embolization group (Figure 1D). There were no significant difference in OS and DFS rates between the two groups after one-to-one propensity score matching (P = 0.951 and P = 0.670, respectively).

**Table 4:** Baseline patient characteristics after propensity score matching

Variable	Embolization Group (n=95)	Non-embolization Group (n=95)	P Value
Age, years*	55.9 (22-80)	56.8 (23-82)	0.582
Sex			0.636
Male	86 (91)	84 (88)	
Female	9 (9)	11 (12)	
Diabetes mellitus			0.449
Yes	19 (20)	15 (16)	
No	76 (80)	80 (84)	
Hypertension			0.698
Yes	15 (16)	17 (18)	
No	80 (84)	78 (82)	
Anti-HCV status			1.000
Positive	4 (4)	4 (4)	
Negative	91 (96)	91 (96)	
HBsAg			1.000
Positive	87 (92)	87 (92)	
Negative	8 (8)	8 (8)	
AFP, ng/mL			0.644
>200	30 (32)	33 (35)	
≤200	65 (68)	62 (65)	
No. of nodules			0.878
Solitary	64 (67)	63 (66)	
Multiple	31 (33)	32 (34)	
Size of main tumor, cm			0.349
>2	80 (84)	75 (79)	
≤2	15 (16)	20 (21)	
Child-Pugh class			0.733
A	90 (95)	91 (96)	
B	5 (5)	4 (4)	
ALT, μ/L			1.000
>40	41 (43)	54 (57)	
≤40	54 (57)	41 (43)	
Platelet count, 10E9/L			0.767
≥100	56 (59)	58 (61)	
<100	39 (41)	37 (39)	

Note: Unless otherwise indicated, data are number of patients, with percentages in parentheses. anti-HCV = antibody to hepatitis C virus, HBsAg = hepatitis B surface antigen. AFP = a-fetoprotein, ALT = alanine aminotransferase,

* Data are medians, and data in parentheses are the range.

### Comparison of OS and DFS rates between the groups with medium-sized (3.1–5.0 cm) HCC

**Figure 2:**
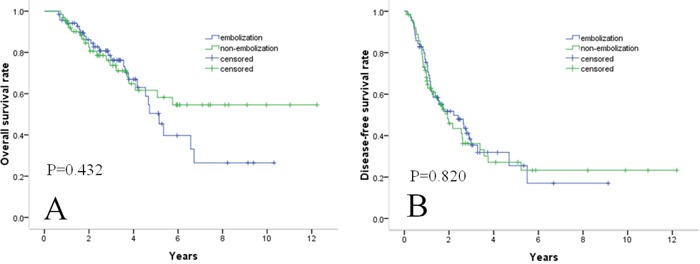
Survival curves in patients with medium-sized (3.1–5.0 cm) HCC OS **A.** and DFS **B.** rates between the two groups did not differ significantly.

Among the 237 study subjects, 70 patients in the embolization group and 64 in the non-embolization group were classified as having medium-sized (3.1–5.0 cm) HCC and were further analyzed. There were no significant differences between the two groups with regard to age, gender ratio, diabetes mellitus, hypertension, hepatitis B surface antigen status, antibody to hepatitis C virus status, AFP levels, Child–Pugh classification, alanine aminotransferase levels, and platelet counts (Table 5). The respective 1-, 3-, and 5-year OS rates were 94%, 76%, and 53% in the embolization group and 95%, 74%, and 62% in the non-embolization group (Figure 2A). The differences between the groups were not significant (P = 0.432). The recurrence rates for medium-sized (3.1–5.0 cm) HCCs were 60% (n = 42) in the embolization group and 60.1% (n = 39) in the non-embolization group. The respective 1-, 3-, and 5-year DFS rates were 75%, 35%, and 26% in the embolization group and 68%, 36%, and 27% in the non-embolization group (Figure 2B). DFS rates between the two groups did not differ significantly (P=0.820).

**Table 5:** Baseline characteristics of patients with medium-sized HCC

Variable	Embolization Group (n=51)	Non-embolization Group (n=51)	P Value
Age, years*	57.7 (32-78)	57.0 (23-82)	0.627
Sex			0.5762
Male	60 (86)	56 (88)	
Female	10 (14)	8 (12)	
Diabetes mellitus			0.290
Yes	16 (23)	10 (16)	
No	54 (77)	54 (84)	
Hypertension			0.334
Yes	18 (26)	12 (19)	
No	52 (74)	52 (81)	
Anti-HCV status			0.337
Positive	1 (1)	0 (0)	
Negative	69 (99)	64 (100)	
HBsAg			0.242
Positive	63 (90)	61 (95)	
Negative	7 (10)	3 (5)	
AFP, ng/mL			0.200
>200	18 (26)	23 (36)	
≤200	52 (74)	41 (64)	
Child-Pugh class			0.143
A	52 (74)	41 (64)	
B	18 (26)	23 (36)	
ALT, μ/L			0.620
>40	32 (46)	32 (50)	
≤40	38 (54)	32 (50)	
Platelet count, 10E9/L			0.925
≥100	41 (59)	38 (59)	
<100	29 (41)	26 (41)	

Note: Unless otherwise indicated, data are number of patients, with percentages in parentheses. anti-HCV = antibody to hepatitis C virus, HBsAg = hepatitis B surface antigen. AFP = a-fetoprotein, ALT = alanine aminotransferase,

* Data are medians, and data in parentheses are the range.

### Complications

There was no treatment-related mortality in either group. In the embolization group, three of 112 patients (2.7%) experienced a major complication. One patient experienced a grade 3 hepatic abscess, requiring percutaneous drainage and antibiotic therapy. A grade 2 pleural effusion was observed in one patient. A grade 2 bile duct stenosis was observed in one patient. In the non-embolization group, three major complications were observed. One patient experienced a grade 2 hepatic abscess, who recovered after antibiotic therapy. A grade 2 intestinal obstruction occurred in one patient, and a grade 2 pneumothorax was observed in another.

## DISCUSSION

TACE and RFA are minimally invasive options that provide the appropriate balance in tumor treatment efficacy and preservation of quality of life [11]. There are several advantages to performing TACE before RFA. First, as the hepatic artery is the primary source of blood supply to an HCC, occlusion of hepatic arterial flow by means of TACE can reduce the cooling effect of the hepatic blood flow on RFA. Therefore, subsequent RFA can induce a bigger area of necrosis [7]. This is beneficial because recurrent tumors commonly occur in the liver remnants near the RFA ablated region [18]. Second, a previous study showed that even when an HCC was solitary and small, micrometastases commonly occurred [19]. Lipiodol and anticancer agents used in TACE can improve the chance of detection and control of invisible micrometastases. Moreover, the heat diffusion within the tumor is perhaps influenced by intratumoral septa and fibrosis. Intratumoral septa usually are disrupted after TACE, which may facilitate heat distribution within the tumor [20]. TACE combined with RFA has been reported to be effective and safe in the treatment of HCC. A recent randomized controlled study has shown that the efficacy of TACE combined with RFA was significantly better than that of RFA alone in patients with HCCs smaller than 7 cm [11]. In a propensity score matching study by Yoshitaka et al. [12], OS rates were comparable between the TACE-RF and surgical resection groups in patients with HCCs within the Milan criteria. Combination therapy with TACE and RF is a highly effective curative treatment for HCC with advantages of an excellent safety rate, rapid wound recovery, and maximal liver function preservation [9–12]. The reported probabilities of overall survival at 1-, 3- and 5-years were 99%, 83% and 58%, respectively, by Yoshitaka et al [12]. In study of Peng et al, the 1-, 3- and 4-year survival rates of patients with a solitary HCC were 79.4%, 60.6% and 54.8%, respectively [11]. The survival rate in our patients who received the combination therapy tended to be lower than that of Yoshitaka et al. and higher than that of Peng et al., although the clinical features were different (etiology, liver function, tumor size, number of tumors and AFP levels).

There has been considerable controversy about the impact of removing embolization to TACE-treated patients’ survival. Hatanaka et al. reported a statistically significant survival benefit of gelatin sponge embolization [16]. In contrast, some studies failed to show survival differences between TACE with or without embolization [14, 15]. In recent studies on combination therapy with TACE and RFA, gelatin sponge-particle embolization after chemolipiodolization was a routine procedure in their protocol [9–13]. However, some clinicians consider gelatin sponge-particle embolization as useless in combination therapy with TACE and RFA. In their opinion, lipiodol can play a sufficient role in occlusion of hepatic arterial flow, for RFA performed within 2 weeks after TACE. In our study, we showed that removal of embolization in combination therapy with TACE and RFA might not significantly decrease OS and DFS rates in patients with HCC within the Milan criteria. Several studies have shown that the combination of TACE and RFA is better for medium-sized (3.1–5.0 cm) [9–10], and we further analyzed these patients. OS and DFS rates between the two groups were still comparable. To the best of our knowledge, our study is the first to address this issue; notably, our study results were obtained before and after balancing patient demographics, tumor characteristics, and liver function between the two groups, which can provide important data with which to optimize guidelines for HCC management. This approach will likely save treatment costs and shorten the operation time if the redundant process of embolization is eliminated.

In our study, both groups had low rates of major complications, and there were no treatment-related deaths. The rate of major complications was 2.7% (3/112 patients) in the embolization group, and 2.4% (3/125 patients) in the non-embolization group. This result was comparable with that in other studies (range, 0.6% – 5.3%) [6, 9–12]. Indeed, all patients with major complications recovered fully after symptomatic treatment, without any serious adverse sequelae. These results suggest that the two TACE-RFA regimens are safe.

Our study had several limitations. First, this study is a retrospective analysis; therefore, outcomes can be attributed to the intervention itself or selection bias. Although we attempted to reduce selection bias by using propensity score analysis, there remained a possibility of uncontrolled potential confounders between the groups. Second, more than 90% of the patients in our study were hepatitis B surface antigen carriers. Therefore, it may be difficult to directly generalize our results to other institutions where hepatitis B viral infection is not the main cause of HCC. Third, we only used gelatin sponge particles in our study; other particles such as polyvinyl alcohol may influence the results of embolization. However, gelatin sponge particles are retained in the tumor for 2 weeks after chemoembolization [21], and RFA followed TACE within 2 weeks.

In summary, embolization in TACE combined with RFA could not improve the survival for patients with HCC within the Milan criteria. Therefore, the removal of embolization from combination therapy with TACE and RFA can be justified considering the low cost and short operation duration. In the future, a multicenter randomized controlled trial should be carried out to further test and to develop the optimal regimen of TACE and RFA in treating HCC.

## MATERIALS AND METHODS

### Patients

In this retrospective cohort study, we reviewed the records of consecutive patients who underwent combination therapy with TACE and RFA as the initial treatment for HCC, from a database that was collected prospectively at two institutions (Sun Yat-sen University Cancer Center and The First Affiliated Hospital of Zhengzhou University) from August 2002 to December 2014. Our study was approved by the institutional review board of each center, and each patient provided informed consent for use of their clinical data. The diagnosis of HCC was based on the diagnostic criteria used by the American Association for the Study of Liver Diseases: two dynamic imaging techniques showing typical features of HCC or positive findings by one imaging technique together with the level of a-fetoprotein (AFP) higher than 200 ng/mL, or histologic diagnosis of HCC. The maximal diameter of the tumors was measured with computed tomography (CT) or magnetic resonance (MR) imaging.

Patient selection was performed using the following inclusion criteria : (a) HCC within the Milan criteria; (b) liver function of Child–Pugh class A or B; (c) performance status less than 2 (22); (d) prothrombin activity above 40% and platelet count of more than 40,000/μL; (e) no previous treatment. Meanwhile, patients who received other kind of first-line treatment and those with simultaneous malignancies were excluded from this study. The final study group comprised 112 patients (median age, 56.7 years; age range, 22–80 years; 97 men, 15 women) in the embolization group and 125 patients (median age, 56.6 years; age range, 23–82 years; 109 men, 16 women) in the non-embolization group. Of these, diagnosis was made with biopsy in 48 patients and with imaging in 189 patients. Patient characteristics are shown in Table 1.

### Treatment

Chemolipiodolization was performed according to the following protocol: Using the Seldinger technique, a selective 5-F catheter (Terumo, Tokyo, Japan) was introduced via a punctured femoral artery. Angiographic survey of the abdominal vessels such as superior mesenteric artery and common hepatic vessels was performed to assess the arterial blood supply to the liver. For tumor treatment, a 2.9-F microcatheter (Terumo Corporation, Tokyo, Japan) was superselectively placed in the feeding arteries of the tumor using a coaxial technique. An emulsion composed of 40–60 mg of epirubicin (Pharmorubicin; Pfizer, Wuxi, China; Farmorubicin; Pharmacia, Tokyo, Japan) and 5–10 mg of mitomycin C (Zhejiang Hisun Pharmaceutical, Taizhou, China; Kyowa Hakko Kogyo, Tokyo, Japan) in 5–10 mL of lipiodol (Lipiodol Ultra-Fluide; Andre’ Guerbet Laboratories, Aulnay-Sous-Bois, France) was slowly injected under fluorescence survey. Pure lipiodol was then injected until the territory of the chemolipiodolized artery showed stagnant flow. For the embolization group, embolization was finally performed with injection of gelatin sponge particles (Hanzhou Alc, Hangzhou, China; 500–1000 μm in diameter) through the microcatheter to reach stasis in the tumor-feeding artery.

RFA was performed within 2 weeks after chemolipiodolization by using a commercially available system (RF 2000; Radio-Therapeutics, Mountain View, CA). After administration of conscious sedation and local anesthesia by an anesthesiologist, the 15-Ga probe-like electrode was inserted into the tumor with ultrasound or CT guidance. After expanded the ten tines of the electrode, the radiofrequency generator was activated with an initial power of 10 W that was increased to 90 W, at the rate of 10 W/min. RFA was applied for 15 min or until the impedance achieved a marked increase. If a marked increase in impedance was not reached, a second application of RF was performed. All the nodules were ablated in one session of RFA. Overlap ablation was performed for tumors measuring more than 3 cm in the longest dimension; with all tumors, the aim was to achieve a sufficiently safe margin of 0.5–1 cm. For assessment of treatment responses and complications, all patients in the RF ablation group underwent contrast–enhanced CT immediately after RF ablation to determine the technical success of the procedure. When the least 0.5-cm hypoattenuation surrounding the entire tumor on both arterial and portal venous phase CT images, the treatment was considered a technical success [23]. Additional session of RFA was performed until complete ablation of the tumor was achieved, if necessary.

### Follow-up

Patients were followed up at the outpatient clinic at 1 month after initial discharge, every 3 months for the first 2 years and then to once every 6 months. At each follow-up visit, blood tests for serum liver function and AFP, and imaging with contrast-enhanced CT was performed. Chest radiography was performed once every 6 months. Tumor recurrence was confirmed with contrast-enhanced MRI, or biopsy if necessary. If there was a possibility of extrahepatic recurrence based on clinical symptoms or unexplained elevation of AFP, chest CT, whole-body bone scintigraphy, and brain MR imaging also were performed. Intrahepatic HCC recurrence was classified as local tumor progression (defined as the appearance of an enhancing tumor at the edge of the ablation zone) and distant intrahepatic recurrence.

When local tumor progression, intrahepatic distant recurrence, or extrahepatic recurrence developed during the follow-up period, second-line treatment such as RFA, surgical resection, TACE, or administration of sorafenib was initiated depending on the recommendations of a multidisciplinary tumor board regarding the number and the site of the tumor recurrence, liver function and general condition of the patient. Overall survival (OS) rate and disease-free survival (DFS) rate were compared between the two groups for evaluation of long-term therapeutic outcomes. The OS rate was calculated from the date of the TACE treatment to either the date of death or the last visit to the hospital before March 1^st^, 2016. DFS was defined as the time during the follow-up period until the patient experienced tumor recurrence or death. Major complications that led to additional therapeutic interventions or prolonged hospitalization were recorded.

### Statistical analysis

Patient characteristics were compared between the embolization and non-embolization groups. Continuous data were analyzed by using the two-sample *t*-tests or Mann–Whitney U test depending on normality of data. Categorical variables were evaluated by using the Chi-square test or Fisher's exact test as appropriate. OS and DFS rates were estimated using the life-table method, and differences in survival rates between the two groups were compared using the log-rank test. Survival curves were estimated using the Kaplan–Meier analyses. Factors potentially influencing OS and DFS were assessed by using Cox proportional hazard models in univariate and multivariate analyses. All potential prognostic factors were entered into the multivariate analysis to assess their significance as independent predictors.

To reduce the effect of potential confounders on selection bias in this retrospective study, propensity score based matching analysis was performed. Independent variables entered into the propensity model included age, sex, diabetes mellitus, hypertension, hepatitis B surface antigen status, antibody to hepatitis C virus status, AFP levels, tumor number, tumor size, Child–Pugh classification, alanine aminotransferase levels, and platelet counts. A 1:1 matching between the groups was accomplished by using the nearest-neighbor matching method with a caliper distance of 0.2 without replacement [24]. After matching, continuous data were analyzed under the assumption of normality by using one-sample *t*-tests or Wilcoxon signed rank tests, and categorical variables were evaluated using the McNemar test. Statistical analyses were performed by using IBM SPSS Statistics 20.0 (IBM Co, Armonk, NY). Statistical tests were two sided. P value less than .05 indicated statistical significance.
